# Novel small molecules downregulate CDK1 expression and inhibit Wnt/β-catenin signaling in cutaneous squamous cell carcinoma by targeting its distinct tumor-specific cellular landscape

**DOI:** 10.1038/s12276-025-01532-y

**Published:** 2025-09-01

**Authors:** Soung-Hoon Lee, Min-Jeong Kang, Mi Ryung Roh, Kang-Yell Choi

**Affiliations:** 1 CK Regeon Inc., Yonsei Engineering Research Park, Yonsei-ro 50, Seodaemoon-gu, Seoul, South Korea; 2 Department of Dermatology, Gangnam Severance Hospital, Cutaneous Biology Research Institute, College of Medicine; Yonsei University, Seoul, South Korea

**Keywords:** Drug development, Squamous cell carcinoma

## Abstract

The Wnt/β-catenin pathway is an attractive target for drug development in various diseases; however, efforts to target it have been limited due to its concerning role in cancer. We previously developed KY19382 and KY19334, small molecules that inhibit the cytosolic function of CXXC-type zinc finger protein 5 (CXXC5), as safe therapeutic agents to restore the suppressed Wnt/β-catenin signaling in several intractable diseases, but the effects of these small molecules on cancer have not been determined. Here, we found that KY19382 and KY19334 inhibited the manifestation of malignant phenotype by inhibiting the Wnt/β-catenin signaling of human cutaneous squamous cell carcinoma (cSCC) cells, which was associated with suppression of cyclin-dependent kinase 1 (CDK1) expression. The induced expression of CDK1 and subsequent Wnt/β-catenin pathway activation were observed in human cSCC patient samples compared with normal samples, and the roles of CDK1 in cellular transformation and Wnt/β-catenin pathway activation in human cSCC cells were validated by CDK1 knockdown. Moreover, the two small molecules attenuated two-stage mouse skin carcinogenesis. Overall, KY19382 and KY19334 could be used as agents to treat cSCC and other types of cancer caused by CDK1 overexpression, as well as diseases caused by cytoplasmic accumulation of CXXC5.

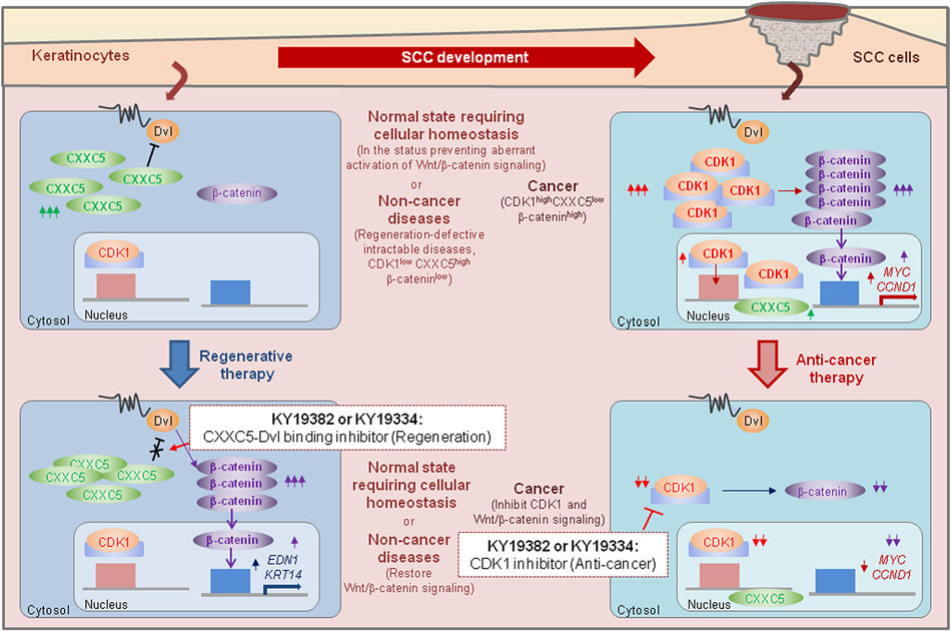

## Small molecules target CDK1 and Wnt pathway to combat skin cancer

Cutaneous squamous cell carcinoma (cSCC) is a common skin cancer with high incidence, prompting the search for new treatments. The study used human cSCC cells and a mouse model to evaluate compounds that regulate Wnt signaling by inhibiting CXXC5 function in diseases caused by reduced Wnt activity. KY19382 and KY19334 were found to inhibit tumor growth by targeting CDK1, a protein essential for cell division. These compounds selectively affected cancer cells without harming normal cells, indicating potential safety. In vivo results confirmed that both agents suppressed tumor development in mice. The researchers concluded that these compounds might offer a new way to treat cSCC by targeting CDK1 without harming normal cells. These findings could lead to the development of safer cancer treatments that specifically target cancerous cells while sparing healthy ones. This summary was initially drafted using artificial intelligence, then revised and fact-checked by the author.

## Introduction

Cutaneous squamous cell carcinoma (cSCC) is the second most frequently occurring skin cancer after basal cell carcinoma and the second leading cause of skin cancer-associated mortality after malignant melanoma^1,2^. Mutations in the skin cancer driver genes such as *HRAS* or topical application of chemicals such as 7,12-dimethylbenzanthracene (DMBA) and 12-*O*-tetradecanoylphorbol-13-acetate (TPA) can result in papillomatous tumors, which are considered as potential progenitor lesions of cSCCs^3,4^. Because cSCC shows a higher incidence than melanoma, developing new therapies for these diseases is important^4,5^.

The Wnt/β-catenin signaling pathway plays critical roles in multiple physiological processes, including proliferation, differentiation and development^6–8^. The aberrant activation of the Wnt/β-catenin pathway by mutations of genes encoding its components such as *APC* and *CTNNB1* plays a role in the tumorigenesis of several types of cancer^9,10^. It also promotes migration, invasion and lymph node metastasis in head and neck squamous cell carcinoma (SCC)^11^. The Wnt/β-catenin pathway is precisely regulated by factors such as CXXC-type zinc finger protein 5 (CXXC5) and cyclin-dependent kinases (CDKs)^12,13^.

We previously demonstrated that the CXXC5, a negative regulator of the Wnt/β-catenin signaling pathway that functions via binding to an upstream Wnt signaling component, Dishevelled (Dvl), is highly expressed in the cytosol of cells in the damaged tissues of patients and the model organisms for several diseases such as androgenetic alopecia, osteoporosis, diabetes, diabetic foot ulcer, metabolic dysfunction-associated steatohepatitis and Alzheimer’s disease^14–17^. The therapeutic approach used in these studies to restore the suppressed Wnt/β-catenin signaling in the tissue cells of several diseases can provide an attractive strategy for safe targeting of the Wnt/β-catenin pathway for drug development^18^. Treatment of the wounded mouse skins with the protein transduction domain-fused Dishevelled binding motif (PTD-DBM) that interrupts the cytosolic CXXC5 function via CXXC5–Dvl protein–protein interaction, or *Cxxc5* knockout showed a regenerative effect on damaged skin tissues through activation of Wnt/β-catenin signaling^12,19,20^. Moreover, co-treatment with the PTD-DBM and valproic acid (VPA), a GSK3β inhibitor, synergistically activates the Wnt/β-catenin pathway and induces skin regeneration^12,19,20^.

KY19382 and KY19334 are the chemically synthesized mimetics of indirubin-3′-oxime (I3O) and 6-bromo-indirubin-3’-oxime (BIO) that have been screened from chemical libraries by using in vitro screening system to monitor CXXC5–Dvl interaction^15,21^. KY19382 and KY19334 restore the Wnt/β-catenin signaling and exert regenerative therapeutic effects by mimicking the function of PTD-DBM in patient tissue cells of hair loss, diabetes, metabolic dysfunction-associated steatohepatitis and Alzheimer’s disease where the Wnt/β-catenin pathway was suppressed by cytosolic CXXC5 overexpression^15–17,22^. Because KY19382 and KY19334 restore the suppressed Wnt/β-catenin signaling by inhibiting cytosolic CXXC5 function, rather than by aberrant activation, they do not induce cancer phenotypes^12,15–17^. Notably, long-term breeding of mice treated with the small molecules as well as *Cxxc5*-knockout mice did not confer the cancer phenotypes^12,20^. Overall, an approach to inhibit cytosolic CXXC5 function can provide a safe way to target the Wnt/β-catenin pathway for drug development^18^. Although the role of cytosolic CXXC5 in the suppression of Wnt/β-catenin signaling and regeneration has been illustrated, its role in cancer cells has not been fully elucidated.

In this study, we demonstrated that KY19382 and KY19334 inhibited the cancer cell phenotypes, including proliferation, migration, invasion and transformation of HSC-1 and HSC-5 cells, human cSCC cells. The anticancer effects of KY19382 or KY19334 are not related to their role in blocking cytosolic CXXC5 function, as indicated by the low levels of CXXC5 in the cytoplasm of cSCC cells. KY19382 and KY19334 attenuated CDK1 expression, as did other indirubin backbone compound I3O^23^, and suppressed Wnt/β-catenin signaling, which is aberrantly activated in human immortalized cSCC cells. To further test the roles of CDK1 and Wnt/β-catenin signaling related to the effects of these compounds in human cSCC, the effect of CDK1 knockdown on transformation and β-catenin activation status was examined in human cSCC cell lines. In addition, the effects of KY19382 and KY19334 on cSCC development as well as CDK1 and Wnt/β-catenin signaling were investigated using a two-stage skin carcinogenesis model. Finally, we further distinguished the roles of cytoplasmic and nuclear CXXC5 in the DMBA/TPA-induced skin carcinogenesis model by measuring the effects of the PTD-DBM, a specific peptide inhibiting the cytosolic function of CXXC5, and *Cxxc5* knockout, respectively.

## Materials and methods

### Ethical approval

To investigate the roles of CDK1, Wnt/β-catenin signaling and CXXC5 in SCC development, the normal (*n* = 3) and SCC (*n* = 5) samples were collected after obtaining informed written consent. Experiments on human samples complied with the World Medical Association Declaration of Helsinki and the Department of Health and Human Services Belmont Report, and were reviewed and approved by the Institutional Review Board of Gangnam Severance Hospital (3-2020-0366). The tissue samples were fixed overnight in 4% (w/v) paraformaldehyde (PFA) for immunohistochemical analyses.

All animal protocols used in this study were approved by the Institutional Review Board of Severance Hospital, Yonsei University College of Medicine (09-013). All procedures related to mice were conducted in compliance with the United Kingdom Animals (Scientific Procedures) Act 1986 and the Animal Research: Reporting of In Vivo Experiments (ARRIVE) guidelines.

### Cell culture and reagents

The human cSCC cells, HSC-1 and HSC-5, were obtained from the Japanese Collection of Research Bioresources. Two human cSCC cells and the control HaCaT cells were cultured in Dulbecco’s modified Eagle medium (DMEM, Gibco) containing 10% (v/v) heat-inactivated fetal bovine serum (Gibco), 100 µg/ml penicillin (Gibco) and 100 µg/ml streptomycin (Gibco) and incubated in 5% (v/v) CO_2_ at 37 °C. For knockdown of CDK1 or CXXC5 expression, the HSC-1, HSC-5 and HaCaT cells were transfected with 100 nM small interfering RNA (siRNA) targeting human CDK1 (Santa Cruz Biotechnology, sc-29252) and CXXC5 (Santa Cruz Biotechnology, sc-91677), respectively using Lipofectamine RNAiMAX Reagent (Invitrogen). KY19382 (5,6-dichloroindirubin-3′-methoxime) and KY19334 (5-methoxyindirubin-3′-oxime) were synthesized in-house^15^. VPA was purchased from Acros.

### Immunocytochemistry

The HSC-1, HSC-5 and HaCaT cells were seeded on glass coverslips in 12-well culture plates at a density of 1 × 10^5^ cells per well. The cells grown on glass coverslips were fixed in 4% (w/v) PFA for 15 min and permeabilized with 0.1% (v/v) Triton X-100 in phosphate-buffered saline (PBS) for 15 min. After washing with PBS three times, the cells were treated with 5% (w/v) bovine serum albumin (BSA) in PBS for 30 min and then incubated with the following primary antibodies: anti-CDK1 (Santa Cruz Biotechnology, sc-54, 1:50), anti-CXXC5 (Lab made, 1:50), anti-CDK4 (Abcam, ab137675, 1:100), anti-HDAC2 (Santa Cruz Biotechnology, sc-9959, 1:50), anti-β-catenin (BD Bioscience, 610154, 1:100), anti-phospho-β-catenin (Ser33/37/Thr41) (Cell Signaling Technology, 9561, 1:100) or anti-p21^Cip/WAF^ (Santa Cruz Biotechnology, sc-397, 1:50) at 4 °C overnight. The cells were washed in PBS three times, followed by incubation with Alexa Fluor 488- (Invitrogen, A-11001, 1:300) or Alexa Fluor 555-conjugated IgG secondary antibody (Invitrogen, A-21428, 1:300) in the dark at room temperature for 1 h. Cell nuclei were counterstained with 4’,6-diamidino-2-phenylindole (DAPI, Sigma-Aldrich, D9564, 1:5,000) for 10 min. The signals were captured with a Nikon Eclipse Ti microscope (Nikon), and quantitative analyses were performed using NIS Elements V3.2 software (Nikon).

### Migration and invasion assays

HSC-1, HSC-5 and HaCaT cells were seeded at a density of 1 × 10^5^ cells per insert in uncoated and Matrigel-coated chambers of 24-well plates to evaluate migration and invasion, respectively. The cells in serum-free DMEM were treated with the indicated compounds, and DMEM containing 10% fetal bovine serum was added to the lower chamber to serve as a chemoattractant. After 24 h incubation, migrating or invading cells were fixed with 4% (w/v) PFA for 15 min and stained with 0.5% (w/v) crystal violet in 20% (v/v) ethanol. The representative images were captured on a bright-field optical microscope (ECLIPSE TE2000-U, Nikon).

### MTT assay

To assess cell proliferation, the HSC-1, HSC-5 and HaCaT cells were plated in 24-well plates at a density of 2 × 10^4^ cells per well. Following cell attachment, the cells were treated with the indicated chemicals or siRNAs. After 72 h of culture, the medium was removed and changed with 300 μl of fresh medium. Subsequently, 15 μl of 3-(4,5-dimethylthiazol-2-yl)-2,5-diphenyltetrazolium bromide (MTT, AMRESCO) reagent was added to each well. After incubation at 37 °C for 1 h, purple formazan crystals were obtained by dissolving 500 μl of dimethyl sulfoxide. The absorbance of formazan at 595 nm was measured on a FLUOstar OPTIMA luminometer (BMG LABTECH).

### Colony formation assay

For colony formation assays, the HSC-1 and HSC-5 cells were seeded into 12-well plates at 200 cells per well, and following cell attachment, the cells were treated with the indicated chemicals. The medium was changed every other day and treated with chemicals at the same concentration. Following 2 weeks of treatment, cells were fixed in 4% (w/v) PFA for 15 min and stained with 0.5% (w/v) crystal violet in 20% (v/v) ethanol for 30 min. The assay was performed in triplicate.

### Animal studies

Six-week-old male C57BL/6 mice (KOATECH) were allowed to acclimate to their new environment for 1 week and used to determine the effect of KY19382 or KY19334 on skin cancer development. The C57BL/6 mice were randomly divided into three groups, and the hairs on 1-cm^2^ dorsal skins of all mice were shaved using a hair clipper to allow topical application of chemicals. All mice were subjected to initiation with single topical application of 15 μg DMBA (Sigma-Aldrich) dissolved in 50 μl acetone, followed by sustained promotion with topical application of 1 μg TPA (Cell Signaling Technology) dissolved in 50 μl acetone three times per week for 24 weeks. Then, 2 mM of KY19382, KY19334 or vehicle was topically applied 30 min before each TPA treatment for 24 weeks. The vehicles for dissolving these two compounds were prepared as previously reported^24^.

Animal experiments testing the effect of PTD-DBM and/or VPA on skin cancer development, were conducted in the same way as the novel CDK1 downregulators were tested (*n* = 10 per group). Using a vehicle consisting of 50% (v/v) ethanol, 30% water and 20% propylene glycol, 2 mM PTD-DBM and 500 mM VPA were prepared. To further investigate the effect of Cxxc5 on skin cancer development, animal experiments using *Cxxc5*^−/−^ mice and their littermate controls were conducted similarly to the previous one (*n* = 10 per group). The *Cxxc5*^−/−^ mice were maintained on the C57BL/6 background as previously described^12^.

### Hematoxylin and eosin (H&E) staining

Skin cancer tissues were collected, fixed overnight in 4% (w/v) PFA in PBS, dehydrated and paraffinized. The tissues were then embedded in paraffin and sliced into 4-μm sections. The slides were deparaffinized by three changes of xylene and rehydrated through a graded ethanol series. The paraffin sections were stained with hematoxylin for 5 min and eosin for 1 min. The stained slides were examined using the bright-field microscope (ECLIPSE TE2000-U, Nikon).

### Immunohistochemistry

For immunofluorescence staining, paraffin sections of skin cancer tissues were deparaffinized and rehydrated. The sections were treated with 10 mM sodium citrate buffer (pH 6.0) and autoclaved for 15 min. The slides were blocked in PBS containing 5% (w/v) BSA at room temperature for 30 min and incubated at 4 °C overnight with the following primary antibodies: anti-β-catenin (BD Bioscience, 610154, 1:100), anti-CDK1 (Santa Cruz Biotechnology, sc-54, 1:50), anti-CXXC5 (Lab made, 1:50), anti-PCNA (Santa Cruz Biotechnology, sc-56, 1:500), anti-c-Myc (Santa Cruz Biotechnology, sc-789, 1:50), anti-Cyclin D1 (Santa Cruz Biotechnology, sc-8396, 1:50), anti-HDAC2 (Santa Cruz Biotechnology, sc-9959, 1:50), anti-Acety-H3 (Millipore, 06-599, 1:200) or anti-p21^Cip/WAF^ (Santa Cruz Biotechnology, sc-397, 1:50). The slides were washed with PBS, followed by incubation with Alexa Fluor 488- (Invitrogen, A-11001, 1:300) or Alexa Fluor 555-conjugated IgG secondary antibody (Invitrogen, A-21428, 1:300) at room temperature for 1 h. The slides were then washed with PBS and counterstained with DAPI (Sigma-Aldrich, D9564, 1:5,000). The stained images were captured using the Nikon Eclipse Ti microscope (Nikon) or LSM700 META confocal microscope (Carl Zeiss).

For 3,3’-diaminobenzidine (DAB) staining, deparaffinization, rehydration and antigen retrieval of the paraffin sections of skin cancer tissues were performed using the same methods used for immunofluorescence. To block endogenous peroxidase activity before DAB staining, the slides were incubated with 1% (v/v) H_2_O_2_ (Samchun Chemicals) at room temperature for 10 min. Mouse IgG was blocked using a MOM Mouse IgG blocking kit (Vector Laboratories) before incubation of sections with mouse primary antibody. The sections were then blocked with PBS containing 5% (w/v) BSA at room temperature for 30 min and incubated with the following primary antibodies: anti-β-catenin (BD Bioscience, 610154, 1:100), anti-PCNA (Santa Cruz Biotechnology, sc-56, 1:500), anti-Cdk1 (Santa Cruz Biotechnology, sc-54, 1:50), anti-Cyclin D1 (Santa Cruz Biotechnology, sc-8396, 1:50), anti-c-Myc (Santa Cruz Biotechnology, sc-789, 1:50), anti-p21^Cip/WAF^ (Santa Cruz Biotechnology, sc-397, 1:50) or anti-p27 (Santa Cruz Biotechnology, sc-1641, 1:50) at 4 °C overnight. The slides were then incubated with biotinylated anti-rabbit (Dako, BA-1000, 1:200) or anti-mouse (Dako, BA-9200, 1:200) secondary antibody at room temperature for 1 h. The samples were incubated in avidin-biotin complex solutions (Vector Laboratories) for 30 min, stained with DAB (Dako) for 2–5 min and counterstained with Mayer’s hematoxylin (Muto). The signals were examined using the bright-field optical microscope (ECLIPSE TE2000-U, Nikon).

### Western blot analyses

Cancerous and noncancerous skin tissues were ground in mortars and lysed in radio-immunoprecipitation assay buffer containing 10 mM Tris–HCl, 5 mM EDTA, 1% (v/v) NP-40, 150 mM NaCl, 20 mM NaF, 1 mM sodium vanadate, 1 mM PMSF and protease inhibitor cocktail (Millipore). Equal amounts of protein were separated on 10–12% (v/v) sodium dodecyl sulfate–polyacrylamide gels and transferred to nitrocellulose membranes (GE Healthcare Life Sciences). Following blocking for 1 h with PBS containing 5% (w/v) nonfat dry skim milk and 0.07% (v/v) Tween 20, the membranes were incubated with the following primary antibodies: anti-β-catenin (Santa Cruz Biotechnology, sc-7199, 1:3,000), anti-active-β-catenin (Millipore, 05-665, 1:1,000), anti-PCNA (Santa Cruz Biotechnology, sc-56, 1:500), anti-c-Myc (Santa Cruz Biotechnology, sc-789, 1:500), anti-Cyclin D1 (Santa Cruz Biotechnology, sc-8396, 1:500), anti-HDAC2 (Santa Cruz Biotechnology, sc-9959, 1:500), anti-Acetyl-H3 (Millipore, 06-599, 1:1,000), anti-p21^Cip/WAF^ (Santa Cruz Biotechnology, sc-397, 1:500) or anti-Erk (Santa Cruz Biotechnology, sc-514302, 1:5,000) at 4 °C overnight. Each membrane was washed three times and then incubated with horseradish peroxidase-conjugated anti-mouse (Cell Signaling Technology, 7076, 1:5,000) or anti-rabbit (Bio-Rad Laboratories, 1706515, 1:5,000) IgG secondary antibody at room temperature for 1 h. The blots were visualized with enhanced chemiluminescence (Amersham Bioscience) using a luminescent image analyzer, LAS-4000 (Fujifilm).

### Statistical analyses

Statistical analyses were performed using GraphPad Prism V6.01 (GraphPad Software). Statistically significant differences were determined using Student’s *t*-tests or one-way analysis of variance. All data are presented as the mean ± standard deviation (s.d.). The statistical significance is expressed in the figures as follows: *P < 0.05, **P < 0.01, ***P < 0.001.

## Results

### KY19382 and KY19334 inhibit the migration, invasion, proliferation and transformation of human cSCC cells

**Fig. 1:**
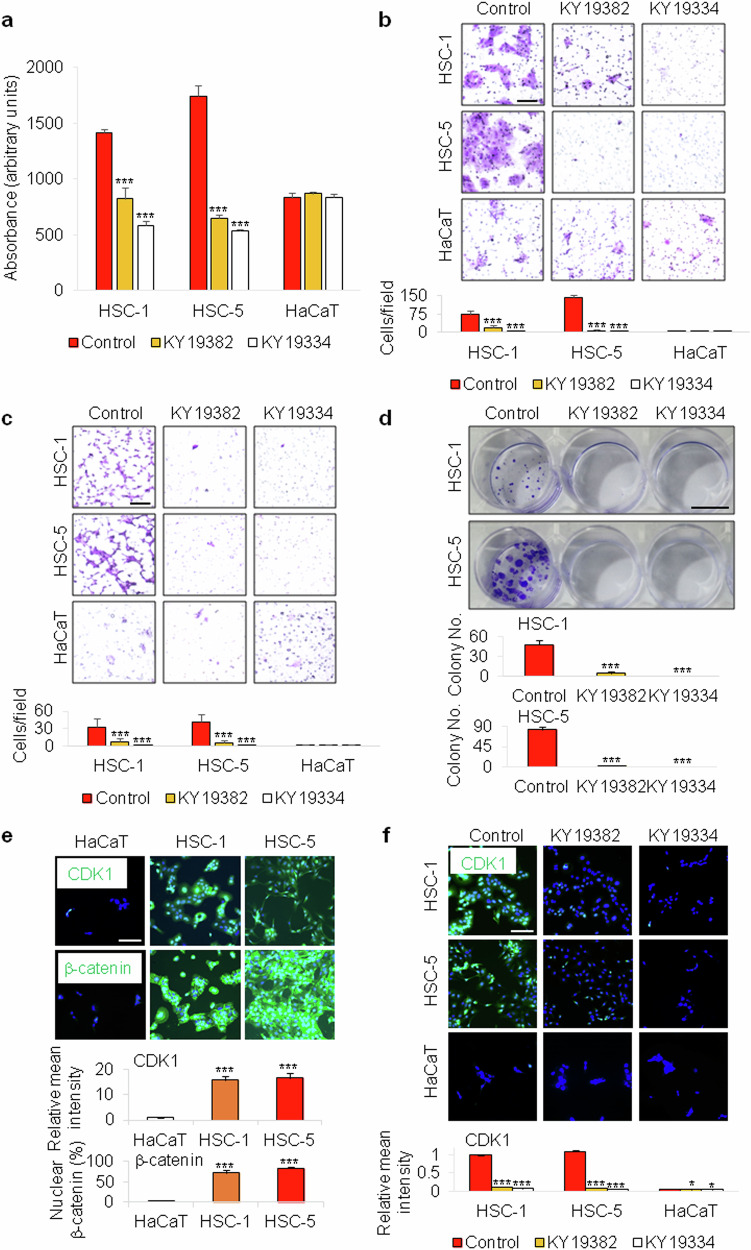
Effects of KY19382 and KY19334 on human cSCC cell lines. **a**, MTT assay (*n* = 3) representing proliferation of HSC-1, HSC-5 and HaCaT cells treated with 5 μM of KY19382 or KY19334 for 72 h. **b**,**c**, Crystal violet staining and its quantitative analyses (*n* = 9) after migration (**b**) and invasion (**c**) assays using human cSCC cell lines and HaCaT cells treated with 5 μM of KY19382 or KY19334 for 24 h. Scale bars, 100 μm. **d**, Representative images of colony formation and their quantitative analyses (*n* = 3) using human cSCC cell lines treated with each indicated compound for 2 weeks. Scale bar, 1 cm. **e**, Immunocytochemical staining for CDK1 (green) or β-catenin (green), DAPI staining (blue) and their quantitative analyses (*n* = 5) in HSC-1, HSC-5 and HaCaT cells. Scale bar, 100 μm. **f**, Immunocytochemical staining for CDK1 (green), DAPI staining (blue) and mean intensity quantitation (*n* = 5) in human cSCC cell lines and HaCaT cells treated with the indicated compound for 24 h. Scale bar, 100 μm. Data are presented as means ± s.d. *P < 0.05, ***P < 0.001 for **a**–**f**.

Our recent studies have shown that KY19382 and KY19334 revealed therapeutic effects on several intractable diseases, which suppress the regenerative characteristics of tissue cells in the disease status through restorative activation of Wnt/β-catenin signaling by disrupting the specific cytosolic CXXC5 function^15,21,22^. To investigate any effect of these compounds on cancer progression, we treated human cSCC cells with KY19382 or KY19334. HaCaT cell line was used as a control for normal keratinocytes^25^. Intriguingly, KY19382 or KY19334 inhibited the proliferation of the two human cSCC cell lines (Fig. 1a). Boyden chamber assay using human cSCC cell lines and a normal keratinocyte cell line also revealed that these two compounds significantly inhibited migration and invasion of the two human cSCC cell lines (Fig. 1b, c). Likewise, KY19382 and KY19334 markedly inhibited the transformation of both HSC-1 and HSC-5 cells (Fig. 1d).

To better understand the inhibitory effects of these compounds on cancer progression, we next monitored the expression levels of cytosolic CXXC5 and CDK1, potential targets of these compounds, and their associated markers in human cSCC cell lines. Differently from normal HaCaT cells, the expression level of CXXC5, especially within the cytoplasm, was very low in both HSC-1 and HSC-5 cells (Supplementary Fig. 1a,b), supporting that cytosolic CXXC5 is not a functional target of the compounds in cSCC cells. The expression of CDK1 was significantly increased in both HSC-1 and HSC-5 cells (Fig. 1e). By contrast, expression of the CDK1 inhibitor p21^Cip/WAF^ (ref. ^26^) was markedly decreased in these cells compared to HaCaT cells (Supplementary Fig. 1a,b). Concomitantly, β-catenin was highly accumulated in both the nucleus and cytoplasm of HSC-1 and HSC-5 cells (Fig. 1e).

In our recent studies, KY19382 and KY19334 were found to upregulate the Wnt/β-catenin pathway through inhibition of the CXXC5-Dvl interaction in noncancerous cells^15,16,22^. However, KY19382 and KY19334 did not activate the Wnt/β-catenin pathway in the human cSCC cell lines, differently from that in HaCaT cells (Supplementary Fig. 2a,b). This might be caused by the low expression levels of cytosolic CXXC5, a functional target of these compounds, in the cytoplasm of HSC-1 and HSC-5 cells compared with HaCaT cells (Supplementary Fig. 1a,b). Intriguingly, these compounds rather downregulated the Wnt/β-catenin pathway in the human cSCC cell lines (Supplementary Fig. 2a,b). Consistently, immunocytochemical analyses revealed that KY19382 and KY19334 increased the levels of phosphorylated β-catenin at Ser33/37/Thr41 in human cSCC cell lines (Supplementary Fig. 3a,b). Given the close relationship between CDK1 and Wnt/β-catenin signaling in cancer cells^13^, the effects of these compounds on CDK1 expression were also investigated in these cells. KY19382 and KY19334 effectively downregulated CDK1 expression, particularly in two human cSCC cell lines (Fig. 1f). By contrast, KY19382 and KY19334 did not alter the expression levels of CDK4, another isoform of the CDK family, as demonstrated by immunocytochemical analyses (Supplementary Fig. 4a,b). Overall, KY19382 and KY19334 inhibit cellular transformation of human cSCC cells, but not normal cells, with suppression of CDK1 expression.

### Increased CDK1 expression and activation of Wnt/β-catenin signaling are observed in human cSCC tissues

**Fig. 2:**
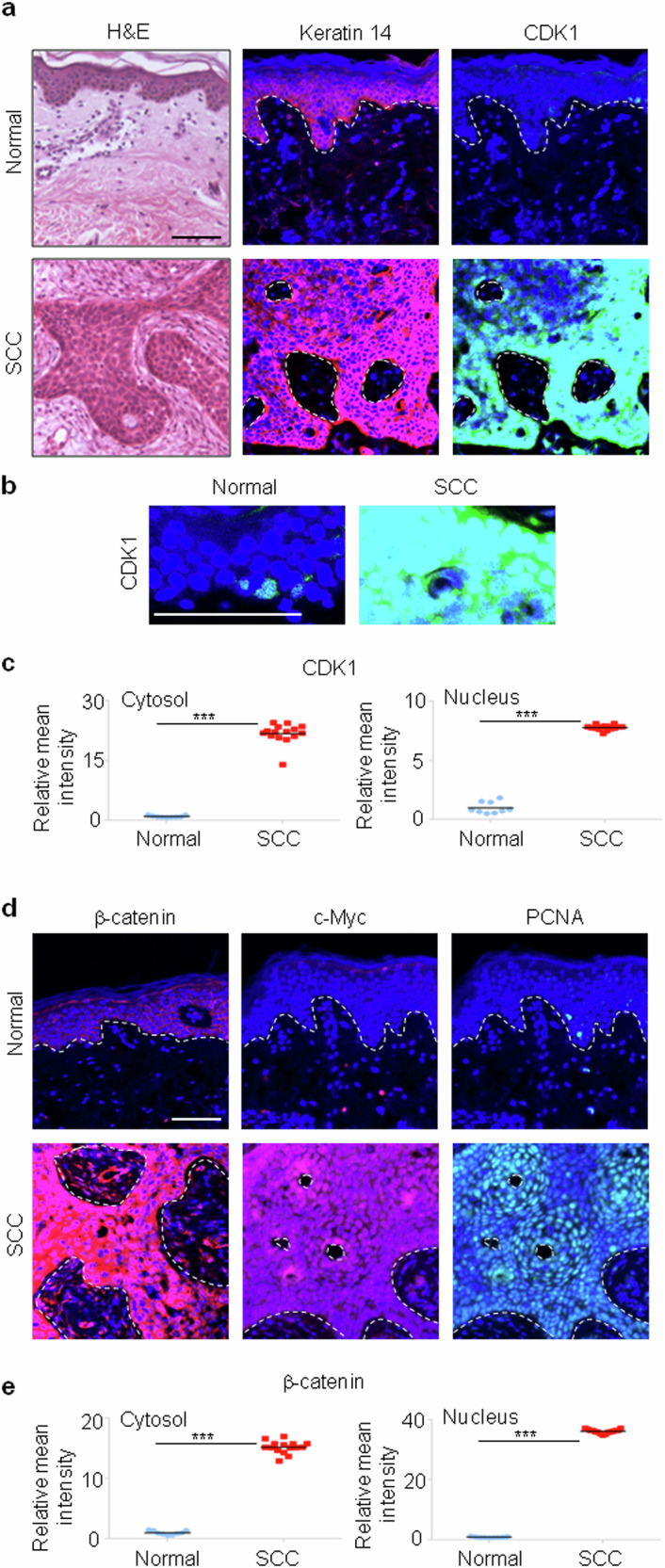
Expression patterns of the CDK1 and β-catenin in human cSCC. **a**, H&E and immunohistochemical staining for Keratin 14 (red) or CDK1 (green) with DAPI nuclear counterstaining (blue) in the human normal skin (*n* = 3) and cSCC (*n* = 5) tissues. Dashed lines indicate the boundaries between K14-positive and K14-negative tissues. Scale bar, 100 μm. **b**, High magnification of CDK1 immunohistochemical staining images with DAPI staining (blue) in the normal skin and cSCC tissues. Scale bar, 100 μm. **c**, Quantitative analyses of immunohistochemical staining with antibody against CDK1. Three representative fields per patient sample were analyzed. **d**, Immunohistochemical staining for β-catenin (red), c-Myc (red) and PCNA (green) with DAPI nuclear counterstaining (blue) in the human normal skin (*n* = 3) and cSCC (*n* = 5) tissues. Dashed lines represent the border between K14-positive and K14-negative tissues. Scale bar, 100 μm. **e**, Quantitative analyses of immunohistochemical staining with antibody against β-catenin. Three representative fields per patient sample were analyzed. Data are represented as means ± s.d. ***P < 0.001 for (**c** and **e**).

To further investigate the clinically relevant functions of CDK1 and Wnt/β-catenin signaling in human skin carcinogenesis, expression of CDK1 and several key Wnt/β-catenin pathway proteins were analyzed in human cSCC patient tissues by immunohistochemical analyses. As reported previously^27^, Keratin 14, a marker for stratified epithelial cells, was expressed in both the epithelium of normal skins and the malignant squamous epithelium (Fig. 2a). Also, the CDK1 expression was significantly higher in the Keratin 14-positive malignant epithelium of cSCC than in the matched normal epithelium (Fig. 2a). Higher-magnification images and quantification showed that CDK1 was sparsely expressed in nucleus in normal skins, whereas CDK1 expression was higher in both the nucleus and cytoplasm in cSCC (Fig. 2b, c). Concurrently, the level of β-catenin was increased in both the nucleus and cytoplasm of cSCC compared with normal (Fig. 2d, e), which is in good correlation with the increased expression of the Wnt/β-catenin signaling targets, c-Myc and PCNA (Fig. 2d). Consistently, both CDK1 and β-catenin expression levels were concurrently upregulated in the well-differentiated squamous epithelium of the papillomatous lesion, compared with the normal epithelium (Supplementary Fig. 5). These results demonstrated that the concurrent increase in CDK1 and β-catenin expression in human cSCC tissues was similar to that observed in two human cSCC cell lines.

### CDK1 knockdown significantly suppresses the cancer cell phenotypes in human cSCC cell lines

**Fig. 3:**
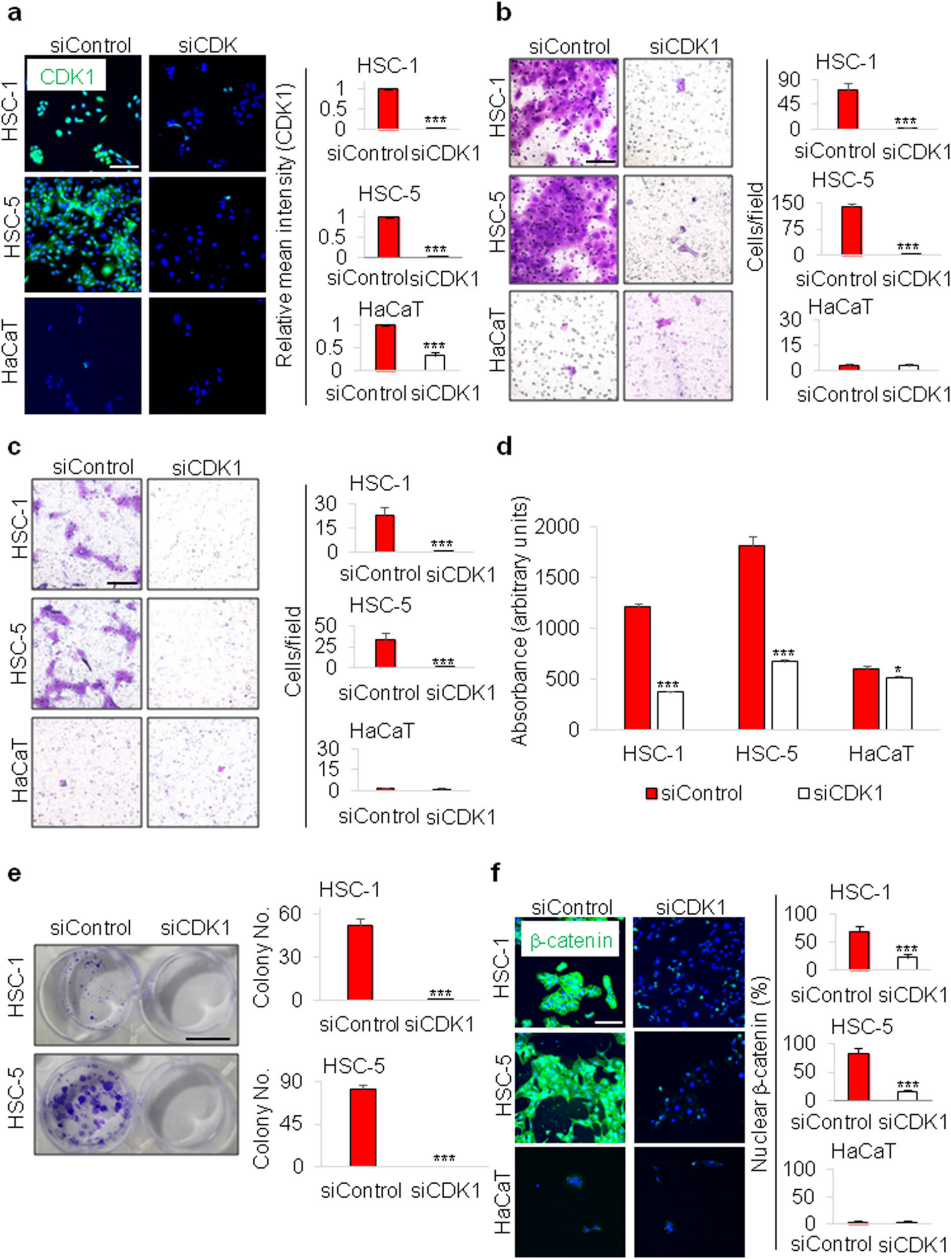
Impacts of CDK1 knockdown on the cancer cell phenotypes in human cSCC cell lines. **a**, Immunocytochemical staining of the HSC-1, HSC-5 and HaCaT cells with antibody against CDK1 (green), DAPI staining (blue) and mean intensity quantitation (*n* = 5). The cells were cultured for 24 h after transfection with 100 nM of control siRNA or CDK1 siRNA. Scale bar, 100 μm. **b**,**c**, Migration (**b**) and invasion (**c**) assays and quantification (*n* = 9) of the migrated or the invaded cells using the collected and the suspended HSC-1, HSC-5 and HaCaT cells transfected with 100 nM of control siRNA or CDK1 siRNA. Scale bars, 100 μm. **d**, MTT assays (*n* = 3) of the HSC-1, HSC-5 and HaCaT cells cultured for 72 h after transfection with 100 nM of control siRNA or CDK1 siRNA. Absorbance indicates the degree of cell proliferation. **e**, Clonogenic assays and quantitative analyses (*n* = 3) of the HSC-1 and HSC-5 cells following transfection with 100 nM CDK1 siRNA. Scale bar, 1 cm. **f**, Immunocytochemical staining of the HSC-1, HSC-5 and HaCaT cells with antibody against β-catenin (green), DAPI staining (blue) and mean intensity quantitation (*n* = 5). The cells were cultured for 24 h after transfection with 100 nM of control siRNA or CDK1 siRNA. Data are expressed as means ± s.d. *P < 0.05, ***P < 0.001 (**a**–**f**).

To determine the roles of CDK1 in human cSCC, HSC cells were transfected with siRNA targeting CDK1 mRNA (Fig. 3a). CDK1 has been reported to critically regulate migration and invasion in cancer cells^13^. Similarly, CDK1 mRNA knockdown potently inhibited migration and invasion in both HSC-1 and HSC-5 cells without any effect in the nontransformed HaCaT cells (Fig. 3b, c). CDK1 mRNA knockdown significantly inhibited the proliferation and transformation characteristics of both cSCC cell lines (Fig. 3d, e).

CDK1 was shown to promote the Wnt/β-catenin signaling in different types of cancer cells such as hepatocellular carcinoma (HCC), breast cancer and lung cancer^13,28^. Therefore, we also tested whether the CDK1 knockdown could affect the Wnt/β-catenin signaling pathway in human cSCC cell lines. The Wnt/β-catenin signaling activity as measured by nuclear β-catenin expression, was critically reduced by CDK1 mRNA knockdown in HSC-1 and HSC-5 (Fig. 3f). However, CDK1 mRNA knockdown did not affect the Wnt/β-catenin pathway in HaCaT cells (Fig. 3f). Overall, the data suggested that CDK1 may be involved in cellular transformation and the activation of Wnt/β-catenin signaling only in cSCC but not in normal keratinocytes.

### KY19382 and KY19334 significantly suppress the DMBA/TPA-induced skin carcinogenesis and markedly reduce both Cdk1 and β-catenin expression in vivo

**Fig. 4:**
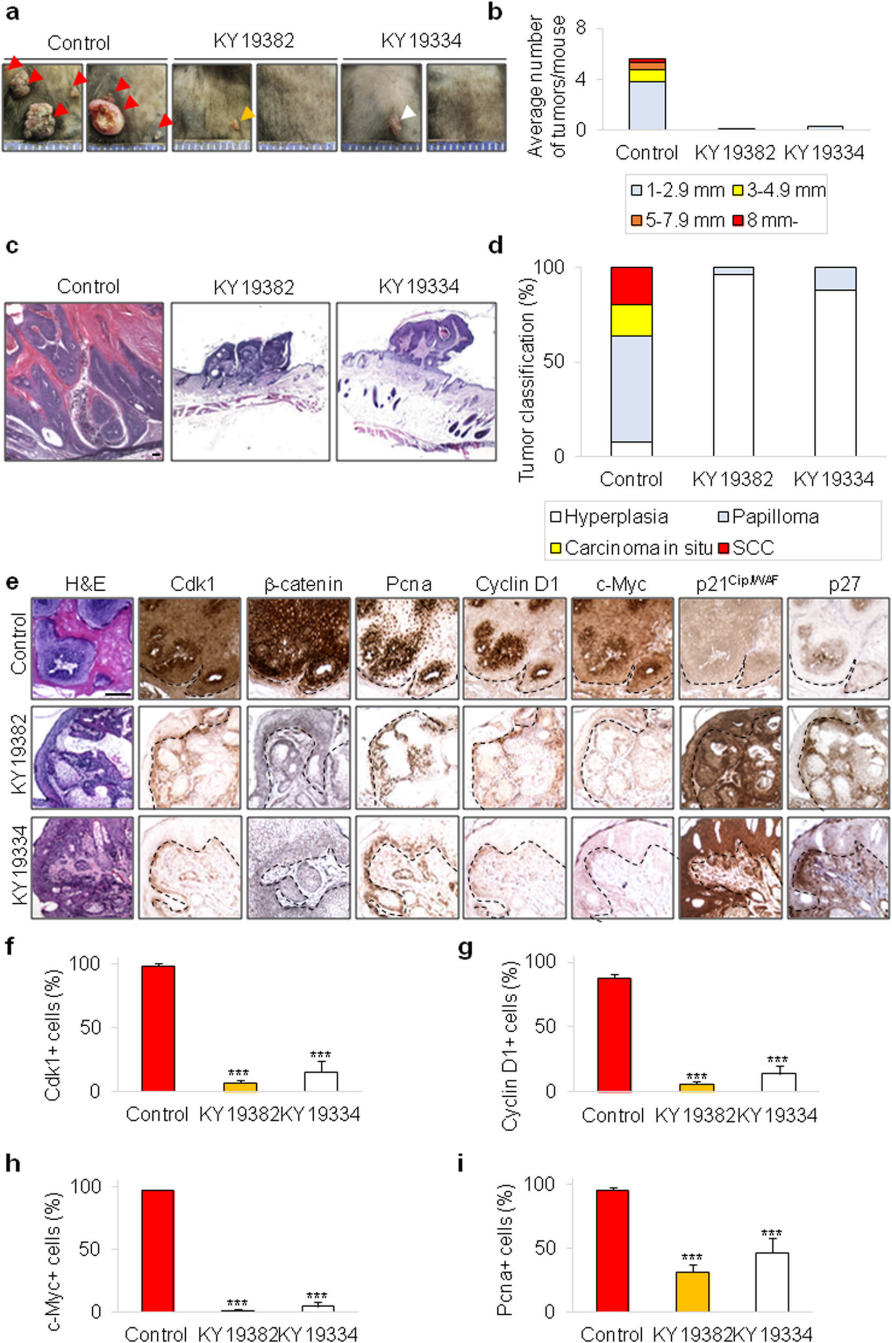
Effects of KY19382 and KY19334 on cSCC development in vivo. **a**, Macroscopic observation of the DMBA/TPA-induced tumors treated with 2 mM of KY19382, KY19334, or vehicle for 24 weeks (*n* = 10 per group). Each arrowhead indicates the position of the tumor. **b**, Quantitative analyses (*n* = 10 per group) of average number of tumors treated with KY19382, KY19334 or vehicle by size. **c**, H&E staining of DMBA/TPA-induced tumors treated with 2 mM of KY19382, KY19334 or vehicle. Scale bar, 100 μm. **d**, Quantitative analyses (*n* = 5 per group) of the tumor grades in mice treated with 2 mM of KY19382, KY19334 or vehicle. **e**, H&E and DAB staining for Cdk1, β-catenin, Pcna, Cyclin D1, p21^Cip/WAF^ or p27 in DMBA/TPA-induced tumors treated with 2 mM of KY19382, KY19334 or vehicle. Scale bar, 100 μm. **f**–**i**, Quantification of Cdk1 (**f**), Cyclin D1 (**g**), c-Myc (**h**) and Pcna (**i**) on the sections stained in **e**. Data are shown as means ± s.d. ***P < 0.001 (**f**–**i**), indicat**i**ng significant differences from the control.

To confirm the effect of pharmacological inhibition of CDK1 by KY19382 and KY19334 on cSCC in vivo, we used the DMBA/TPA-induced two-stage skin carcinogenesis model. Gross image and quantitative analysis showed that both the size and number of DMBA/TPA-induced tumors were greatly reduced by KY19382 or KY19334 (Fig. 4a, b). Treatment with KY19382 or KY19334 significantly lowered the multiplicity of papillomas as well as the tumor incidence (Fig. 4b). Histological and quantitative analyses further revealed that KY19382 and KY19334 markedly attenuated the development and progression of skin tumors (Fig. 4c, d). To further identify changes in the molecular signatures of skin cancer by treatment with KY19382 or KY19334 in vivo, we detected several markers related to oncogenesis in DMBA/TPA-induced tumors. Both KY19382 and KY19334 dramatically reduced Cdk1 levels in DMBA/TPA-induced tumors as in human cSCC cell lines (Fig. 4e, f). Concomitantly, the level of β-catenin was significantly decreased in the skin tumor by either KY19382 or KY19334 (Fig. 4e). Treatment with KY19382 or KY19334 also significantly downregulated expression levels of c-Myc and Cyclin D1, indicating that these compounds reduced the oncogenesis of skin cancer through inhibition of the Wnt/β-catenin pathway (Fig. 4e, g, h). The level of proliferating cell nuclear antigen (PCNA), a proliferation marker, was markedly reduced in KY19382- or KY19334-treated skin cancers (Fig. 4e, i). Furthermore, p21^Cip/WAF^ and p27, tumor suppressors inhibiting the CDK1, were significantly increased by treatment with KY19382 or KY19334 in DMBA/TPA-induced tumors (Fig. 4e). Overall, KY19382 and KY19334 markedly suppressed the development and progression of cSCC with simultaneous inhibition of CDK1 expression and subsequent Wnt/β-catenin pathway activation both in vitro and in vivo.

### Cytoplasmic CXXC5 is not associated with the development and progression of cSCC

**Fig. 5:**
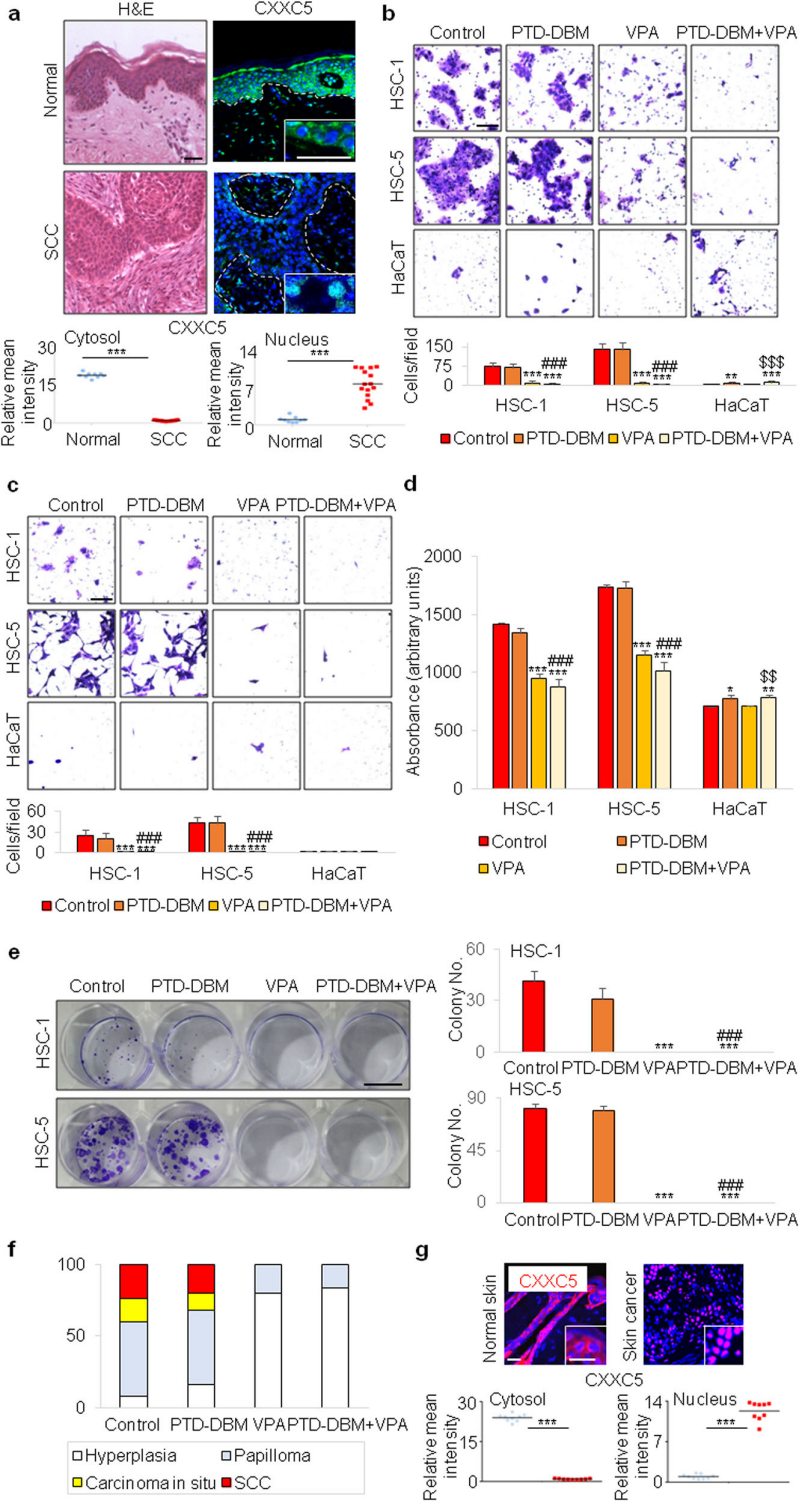
Effects of disruption of CXXC5–Dvl interaction on cSCC in vitro and in vivo. **a**, H&E, immunohistochemical staining for CXXC5 (green) with DAPI nuclear counterstaining (blue) and its quantitation in the human normal skin (*n* = 3) and cSCC (*n* = 5) tissues. The dashed lines demarcate K14-positive and negative tissues, and the inset images show the enlarged images. Three representative fields per patient sample were analyzed. Scale bar, 50 μm. **b**,**c**, Crystal violet staining and quantitative analyses (*n* = 9) after migration (**b**) and invasion (**c**) assays using HSC-1, HSC-5 and HaCaT cells treated with 10 μM PTD-DBM and/or 2.5 mM VPA for 24 h. Scale bars, 100 μm. **d**, MTT assay (*n* = 3) showing proliferation of HSC-1, HSC-5 and HaCaT cells treated with 10 μM PTD-DBM and/or 2.5 mM VPA for 72 h. **e**, Representative images of cell colonies stained with crystal violet and quantitative analyses (*n* = 3) using HSC-1 and HSC-5 cells treated with 10 μM PTD-DBM and/or 2.5 mM VPA for 2 weeks. Scale bar, 1 cm. **f**, Quantitative analyses (*n* = 5 per group) of the histopathological grades identified in DMBA/TPA-induced tumors treated with 2 mM PTD-DBM and/or 500 mM VPA for 24 weeks. **g**, Immunohistochemical staining for Cxxc5 (red) and its quantitation in the normal skin and skin cancer tissues of mice. The inset images represent the enlarged images. Data are represented as means ± s.d. *P < 0.05, **P < 0.01, ***P < 0.001 versus the control group; ###*P* < 0.001 versus the PTD-DBM treatment group; $$*P* < 0.01, $$$*P* < 0.001 versus the VPA treatment group for **a**–**g**.

We next investigated the role of cytoplasmic CXXC5, an inhibitor of Wnt/β-catenin signaling in normal cells and a major target of KY19382 and KY19334^19,22^, in skin carcinogenesis. The expression level and subcellular localization of CXXC5 were first analyzed in human cSCC patient samples. The cytoplasmic CXXC5 was expressed in human normal epithelium, whereas its cytoplasmic expression was greatly reduced in human cSCC (Fig. 5a). Intriguingly, the CXXC5 was expressed in the nucleus in malignant epithelium of cSCC, as shown in the high-magnification images and quantification (Fig. 5a). The result is consistent with the previous reports indicating that CXXC5 acts as either a negative regulator of regeneration by primarily binding to Dvl in the cytoplasm of normal cells^12,19^ or as an oncogene in the nucleus of cancer cells^29,30^. The differential localization of CXXC5 observed in normal skin and cSCC cells suggests that CXXC5 plays distinctive roles in normal and cancer tissues.

We previously identified that the PTD-DBM peptide restores the Wnt/β-catenin pathway by interfering the CXXC5–Dvl interaction that suppresses this pathway in the cells of several disease tissues^12,19,20^. To further determine the function of cytoplasmic CXXC5 in the development and progression of cSCC, in this study, we examined the effect of PTD-DBM, which inhibits specific cytosolic CXXC5 function, on the transformation of cSCC cells. Although the PTD-DBM could induce migration and proliferation of HaCaT cells, it did not affect cancer cell phenotypes of HSC-1 and HSC-5 cells (Fig. 5b–e). Moreover, the PTD-DBM did not alter the DMBA/TPA-induced development of skin tumors (Fig. 5f and Supplementary Fig. 6a,b), and it did not alter the expression of oncogenic markers in both noncancerous and cancerous mouse skin tissues (Supplementary Fig. 6c,d). These data raised the possibility that the levels of cytosolic CXXC5, a target of PTD-DBM action, may not affect cellular transformation and DMBA/TPA-induced tumorigenesis. Accordingly, we investigated the expression level and localization of Cxxc5 in normal skin and skin cancer tissue cells by immunohistochemical analyses. Cxxc5 was translocated from the cytosol to the nucleus in skin cancer tissue cells by DMBA/TPA treatment (Fig. 5g). This result is consistent with the finding that PTD-DBM does not affect cell transformation and DMBA/TPA-induced tumorigenesis in skin tissue. Wnt signaling appears to be unaffected by PTD-DBM due to the absence of cytosolic CXXC5 in the cancer tissue cells (Fig. 5g and Supplementary Fig. 6c).

We have previously used VPA, which directly activates Wnt/β-catenin signaling through GSK3β inhibition, together with PTD-DBM to synergistically activate the Wnt/β-catenin pathway and induce regeneration in the skin tissues^12,19^. VPA did not show a cumulative effect on Wnt/β-catenin activation after its long-term treatment in the normal skin, and slightly reduced Wnt/β-catenin signaling with inhibition of tumorigenesis in the DMBA/TPA-induced skin cancer model (Supplementary Fig. 6c). VPA treatment is also known to induce proteasome degradation of histone deacetylase 2 (HDAC2) in human and mouse carcinoma cell lines^31^. Consistently, immunocytochemical and quantitative analyses using the two human cSCC cell lines showed that the expression of HDAC2 was critically decreased in the group treated with VPA alone or with PTD-DBM and VPA (Supplementary Fig. 7a,b). In addition, VPA significantly inhibited the development and progression of cSCC, but the PTD-DBM had no additional effects on the inhibition of cellular transformation and chemical-induced tumorigenesis by VPA both in vitro and in vivo (Fig. 5b–f and Supplementary Fig. 6). Collectively, long-term treatment with the PTD-DBM reveals no effect on cSCC development, suggesting that the CXXC5–Dvl interaction, the negative feedback mechanism of Wnt/β-catenin signaling, is not involved in cSCC development due to lack of cytosolic Cxxc5 expression.

### CXXC5 is localized in the nucleus in cSCC, and inhibition of CXXC5 expression reveals a mild anticancer effect in cSCC

**Fig. 6:**
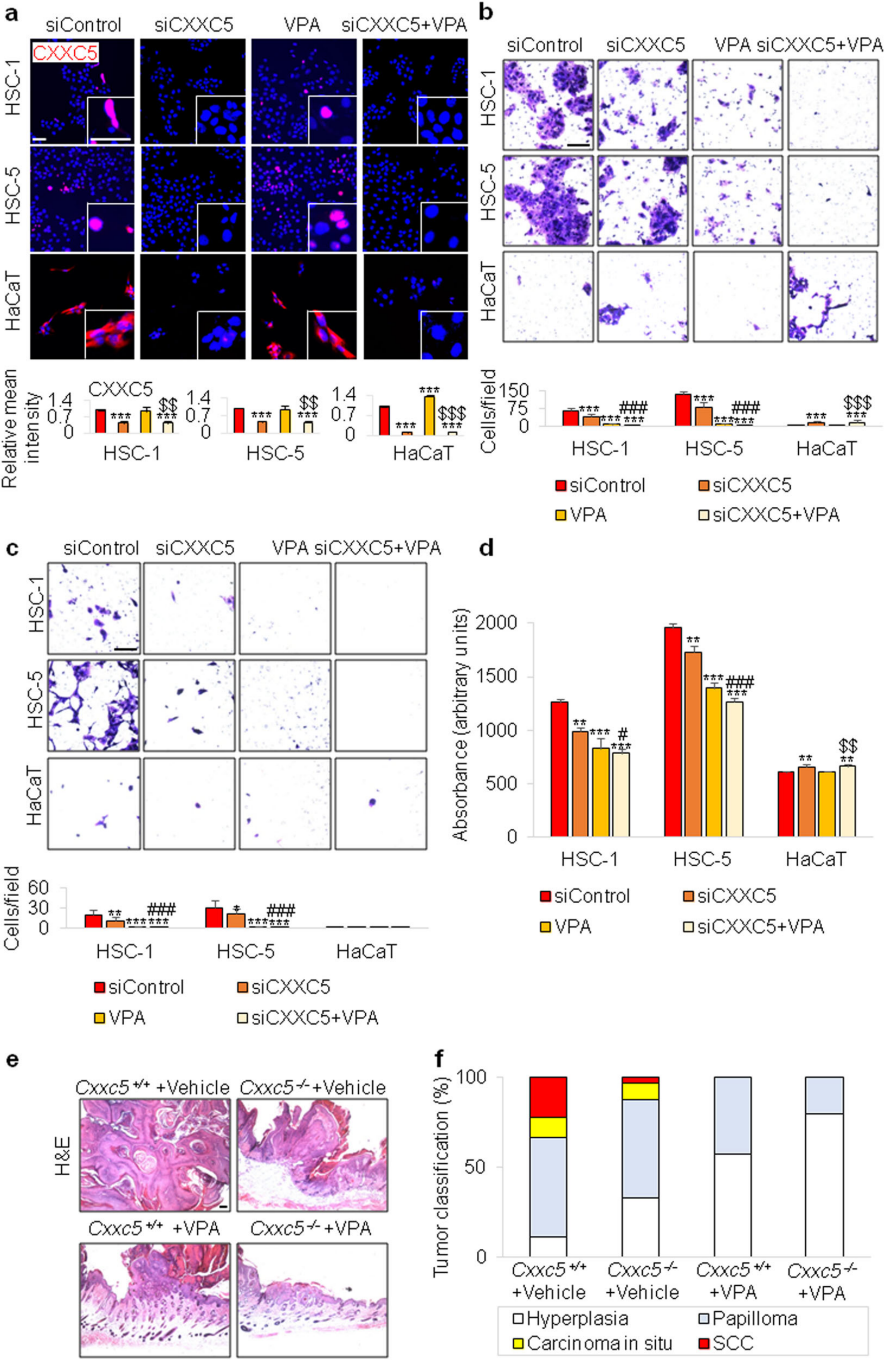
Influence of reduced CXXC5 expression on cSCC in vitro and in vivo. **a**, Immunocytochemical staining with antibody against CXXC5 (red), DAPI staining (blue) and mean intensity quantitation (*n* = 5) of the HSC-1, HSC-5 and HaCaT cells with or without further treatment with 2.5 mM VPA after transfection with 100 nM of control siRNA or CXXC5 siRNA. Insets show CXXC5-stained images at higher magnification. Scale bar, 50 μm. **b**,**c**, Migration (**b**) and invasion (**c**) assays, and quantification (*n* = 9) of migrated or invaded cells using collected and suspended HSC-1, HSC-5 and HaCaT cells treated with 2.5 mM VPA and/or 100 nM CDK1 siRNA. Scale bars, 100 μm. **d**, MTT assay (*n* = 3) using HSC-1, HSC-5 and HaCaT cells treated with or without 2.5 mM VPA for 72 h following transfection with 100 nM CXXC5 siRNA. **e**, H&E staining of DMBA/TPA-induced tumors arising in *Cxxc5*^+/+^ and *Cxxc5*^−/−^ mice treated with or without 500 mM VPA for 24 weeks (*n* = 10 per group). Scale bar, 100 μm. **f**, Quantitative analyses (*n* = 5 per group) of the histopathological grades identified in DMBA/TPA-induced tumors arising in *Cxxc5*^+/+^ and *Cxxc5*^−/−^ mice treated with or without 500 mM VPA for 24 weeks. Data are represented as means ± s.d. *P < 0.05, **P < 0.01, ***P < 0.001 versus the control group; $$*P* < 0.01, $$$*P* < 0.001 versus the VPA treatment group; #*P* < 0.05, ###*P* < 0.001 versus the CXXC5 knockdown group for **a**–**d**.

One can raise the question on the role of CXXC5 in cancerous tissue, considering the changes in CXXC5 localization in skin cancer. Differently from its cytosolic localization in normal cells, CXXC5 is expressed in the nucleus of cancer cells. The nuclear CXXC5 is known to be a poor prognostic factor in a variety of cancers, including human malignant peripheral nerve sheath tumor, prostate cancer, thyroid cancer, breast cancer and metastatic melanoma^30,32^. The CXXC5 was highly expressed in the cytoplasm of normal HaCaT cell lines, whereas it was expressed in the nucleus of HSC-1 and HSC-5 cancer cell lines (Fig. 6a). To further investigate the role of CXXC5 in human cSCC, we examined the effect of CXXC5 knockdown on HSC-1 and HSC-5 cells. CXXC5 was hardly detected in both normal and cancer cells after CXXC5 knockdown (Fig. 6a). As reported previously on the role of CXXC5 in cancer cells^29,33^, CXXC5 knockdown weakly inhibited the migration and invasion of HSC-1 and HSC-5 cells (Fig. 6b, c). Knockdown of CXXC5 expression regulated proliferation differently in normal keratinocytes and human cSCC cells, suggesting a differential role of CXXC5 in normal and cancer cells based on their different subcellular localization (Fig. 6d). Moreover, CXXC5 knockdown reduced the transformation of HSC-1 cells (Supplementary Fig. 8a,b). We also investigated the effect of CXXC5 knockdown on Wnt/β-catenin pathway activation. As monitored by nuclear β-catenin quantification, Wnt/β-catenin signaling activity was low in normal HaCaT cells and increased by CXXC5 knockdown in these cells. However, Wnt/β-catenin signaling was not modulated by CXXC5 knockdown in HSC-1 and HSC-5 cells (Supplementary Fig. 9a,b). This result is opposite to the effect of CDK1 knockdown on the Wnt/β-catenin pathway in normal keratinocyte and cSCC cell lines (Fig. 3f).

To determine the effect of *Cxxc5* deletion on carcinogenesis in vivo, *Cxxc5*^−/−^ mice and their littermate controls were subjected to DMBA/TPA-induced skin carcinogenesis. Ablation of Cxxc5 in mice reduced the size and number of DMBA/TPA-induced tumors (Supplementary Fig. 10a). Histological analyses and the quantification in *Cxxc5*^−/−^ and control mice also revealed that *Cxxc5* knockout inhibited skin cancer development in the mice (Fig. 6e, f). Consistently, as demonstrated by immunohistochemical and western blot analyses, oncogenic markers such as c-Myc and Cyclin D1^34^ as well as Pcna were moderately reduced in skin cancers of *Cxxc5*^−/−^ mice (Supplementary Fig. 10b,c). Together with the results from the PTD-DBM treatment showing a role for cytoplasmic CXXC5 in skin carcinogenesis, these findings suggest that nuclear CXXC5 may play an oncogenic role in cSCC and is independent of Wnt/β-catenin signaling.

We previously demonstrated that knockdown or knockout of CXXC5 in combination with treatment with a Wnt/β-catenin signaling activator such as VPA synergistically activates the Wnt/β-catenin pathway in normal skin tissues^12,19^. Based on this finding, we treated human cSCC cell lines with both CXXC5 siRNA and VPA. Intriguingly, VPA alone markedly suppressed migration, invasion, proliferation and transformation of human cSCC cell lines (Fig. 6b–d and Supplementary Fig. 8a,b). Moreover, VPA effectively attenuated skin carcinogenesis induced by DMBA/TPA in mice (Fig. 6e, f and Supplementary Fig. 10a–c). The anti-transforming effect of VPA is not related to the inhibition of GSK3β, which activates Wnt/β-catenin signaling and has a synergistic effect with CXXC5-Dvl binding inhibitors such as PTD-DBM^35,36^. VPA exerts different effects depending on the cell type^35,37^. VPA induces hair regeneration and cutaneous wound healing in mice by activating the Wnt/β-catenin pathway in nontumorous tissues^35,36^. Meanwhile, VPA inhibited proliferation and induced apoptosis in human melanoma cells by upregulating histone acetylation and p21^Cip/WAF^ expression^37^. The anticancer effects of VPA are acquired by its role as a HDAC inhibitor, as confirmed by the critical inhibition of HDAC2 expression as well as the increment of acetyl-H3^38^ (Supplementary Fig. 6c,d). The effects of *Cxxc5* knockout and/or VPA treatment on skin carcinogenesis demonstrate that approaches targeting CXXC5 may be a safe way to target the Wnt/β-catenin pathway. Overall, KY19382 and KY19334, which have advantages as regenerative therapeutic agents in diseases with high levels of cytosolic CXXC5, could potentially be developed as anticancer agents to treat cSCC with high levels of CDK1 expression.

## Discussion

Recently, we found that two small molecules, KY19382 and KY19334, exhibit regenerative therapeutic effects in several intractable diseases characterized by impaired regenerative capacity due to suppression of the Wnt/β-catenin pathway by cytosolic accumulation of CXXC5^14–17,19^. The two small molecules restoratively activated the suppressed Wnt/β-catenin signaling in the disease-status tissue cells via specific inhibition of cytosolic CXXC5 function by interfering with its binding to the upstream component Dvl^14,15,17,22^.

In this study, we found that KY19382 and KY19334 suppressed multiple cancer cell phenotypes both in vitro and in vivo. Both established human SCC cells and SCC patient tissue cells showed high Wnt/β-catenin signaling activity, and this was inhibited by KY19382 and KY19334. The inhibitory roles of these compounds on Wnt/β-catenin signaling and cellular transformation are not related to their role in inhibiting CXXC5 function, as indicated by the absence of cytosolic CXXC5 in cSCC cells. The independence of cytosolic CXXC5 from the anticancer effects of these compounds was further confirmed by the absence of any effect of PTD-DBM, a peptide that specifically interferes with cytoplasmic CXXC5 function, on the transformation of cSCC cells. As other indirubin-based compounds such as I3O^23^, KY19382 and KY19334 also attenuated CDK1 expression. The inhibition of Wnt/β-catenin signaling activity by the compound was caused by downregulation of CDK1 expression, as confirmed by CDK1 knockdown in human cSCC cell lines. The mechanism of CDK1 downregulation and subsequent inactivation of Wnt/β-catenin signaling by KY19382 and KY19334 was further supported by the results showing that these compounds decreased cyclin D1 and c-Myc, two major transcriptional targets of this signaling. The tumor-suppressing function of the compounds by inhibition of the CDK1 and Wnt/β-catenin signaling was demonstrated by their role in inhibiting the cell cycle regulators p21^Cip/WAF^ and p27 with suppression of the DMBA/TPA-induced skin carcinogenesis. The anti-transformation effect of KY19382 and KY19334 is limited to cancer cells harboring an aberrantly activated Wnt/β-catenin signaling pathway by increased CDK1 expression. Therefore, these compounds had no inhibitory effect on normal cells that maintained low levels of CDK1. Overall, KY19382 and KY19334 reveal therapeutic effects on the diseases, requiring regeneration of damaged tissue cells and cancer through opposing regulation of Wnt/β-catenin pathway, by targeting cytosolic CXXC5 and CDK1, respectively.

The regenerative therapies targeting CXXC5, especially those targeting cytosolic CXXC5, may provide a unique opportunity to treat intractable diseases with lowered regenerative characteristics through restorative activation of the Wnt/β-catenin signaling, and these therapies are not related to the role of CXXC5 as an oncogene in the nucleus^29,30,32^. In addition, the current anticancer effect targeting CDK1 by KY19382 and KY19334 does not engage CXXC5, as supported by our analyses revealing the role of nuclear CXXC5 in skin carcinogenesis.

**Fig. 7:**
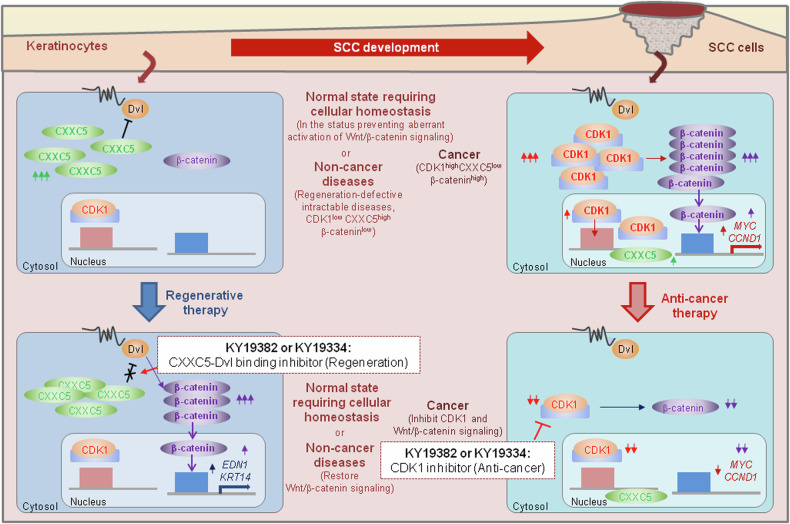
Schematic models depicting the differential effects of KY19382 and KY19334 on normal keratinocytes and SCC cells. Schematic image representing the mechanism of action of KY19382 and KY19334 in noncancer and cancer cells. Left: in the tissue cells of normal state or noncancer diseases, Wnt/β-catenin signaling is suppressed by cytosolic overexpression of the negative regulator CXXC5. Especially, the normal state in which CXXC5 is increased includes stages requiring homeostasis, such as the late stage of cutaneous wound healing or the regression phase of hair follicle cycling^12,19^. The specific inhibition of the cytosolic function of CXXC5 by its interaction with Dvl by KY19382 or KY19334 restores the suppressed Wnt/β-catenin signaling. This homeostasis process is similar to the restoration of suppressed Wnt/β-catenin signaling in the disease-status cells by the compounds. This results in the expression of target genes that are important for the regeneration of specific tissue cells, such as *EDN1*, or are required for various normal cellular functions, such as *KRT14*. Right: in skin cancer, Wnt/β-catenin signaling is activated by increased CDK1 expression. The activation of Wnt/β-catenin signaling induces the expression of oncogenic genes such as *MYC* and *CCND1*. The inhibition of CDK1 by KY19382 or KY19334 results in inactivation of Wnt/β-catenin signaling with repression of c-Myc and cyclin D1.

Our studies illustrated that the differential therapeutic effects of KY19382 and KY19334 depending on the different pathological statuses of cells may enable the potential application of these compounds for regenerative or anticancer therapies (Fig. 7). In addition, in the normal-state cells, CXXC5 may play a role in tissue homeostasis after replacement of the damaged tissue cells by physiological events, such as the late stage of wound healing of skin tissue or the regression phase of hair follicle cycling^12,19^. This homeostasis process is similar to the suppression of Wnt/β-catenin signaling in the disease-status cells, which suppressed Wnt/β-catenin signaling by cytosolic CXXC5^39,40^. In conclusion, the differential regulation of Wnt/β-catenin signaling by the two small molecules targeting cytosolic CXXC5 and CDK1 in noncancerous and cancerous tissue cells, respectively, may help treat both chronic diseases and cancer.

## Supplementary information

Supplementary Information

## Data Availability

The datasets analyzed during the current study are available from the corresponding author upon reasonable request.
