# Supplementary material for: Novel small molecules downregulate CDK1 expression and inhibit Wnt/β-catenin signaling in cutaneous squamous cell carcinoma by targeting its distinct tumor-specific cellular landscape
Source: Exp Mol Med. 2025 Sep 1;57(9):1996–2009. doi: 10.1038/s12276-025-01532-y (PMC12508097; doi:10.1038/s12276-025-01532-y)
Supplement: Supplementary file 1 — Supplementary Information [file 12276_2025_1532_MOESM1_ESM.pdf]

## SUPPLEMENTARY FIGURES AND FIGURE LEGENDS

# Novel small molecules downregulate CDK1 expression and inhibit Wnt/β-catenin signaling in cutaneous squamous cell carcinoma by targeting its distinct tumor-specific cellular landscape

Soung-Hoon Lee[1✉], Min-Jeong Kang[1], Mi Ryung Roh[2], and Kang-Yell Choi[1,3✉]

[1]CK Regeon *Inc*., Engineering Research Park, Yonsei University, Seoul, 03722, South Korea.

[2]Department of Dermatology, Gangnam Severance Hospital, Cutaneous Biology Research Institute, College of Medicine; Yonsei University, Seoul, 06273, South Korea.

[3]College of Life Science and Biotechnology, Building 122, Yonsei University, Seoul, 03722, South Korea.

✉email: greateondal84@yuhs.ac; kychoi@yonsei.ac.kr

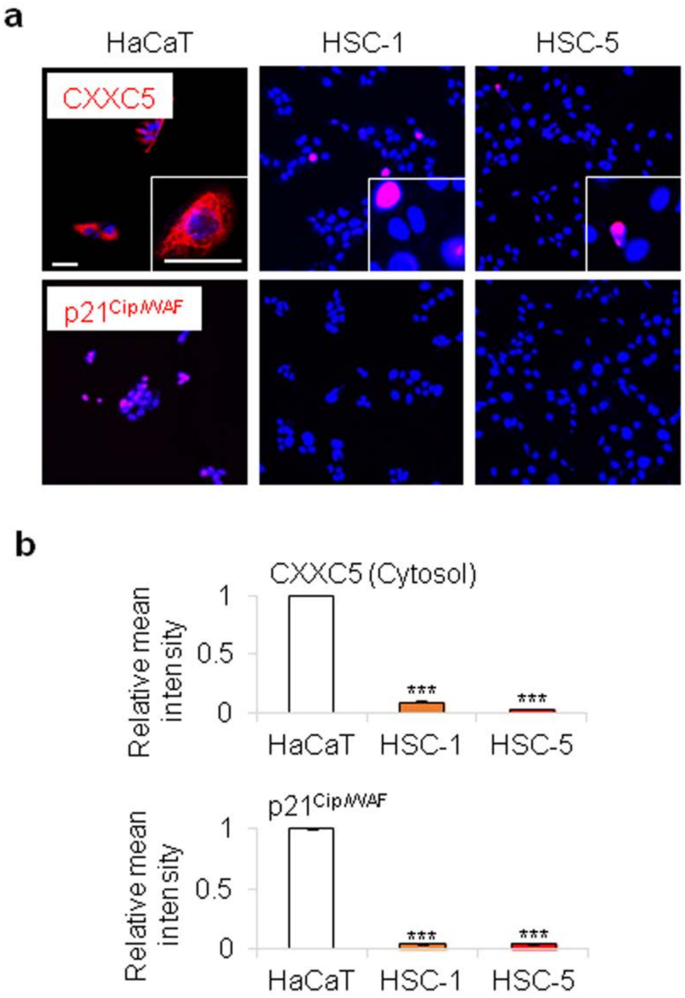


**Supplementary Fig. 1 Molecular characteristics of human cSCC cell lines. a** Immunocytochemical staining for CXXC5 (red) or p21$^{Cip/WAF}$, and DAPI staining (blue) in HCS-1, HCS-5, and HaCaT cells. Insets showed CXXC5-stained images at higher magnification. Scale bar, 50 μm. **b** Quantification ($n$ = 5) of cytosolic CXXC5 and p21$^{Cip/WAF}$ in the cells stained in Supplementary Fig. 1a. Data are represented as means ± SD. ***$P$ <0.001 for panel **b**.

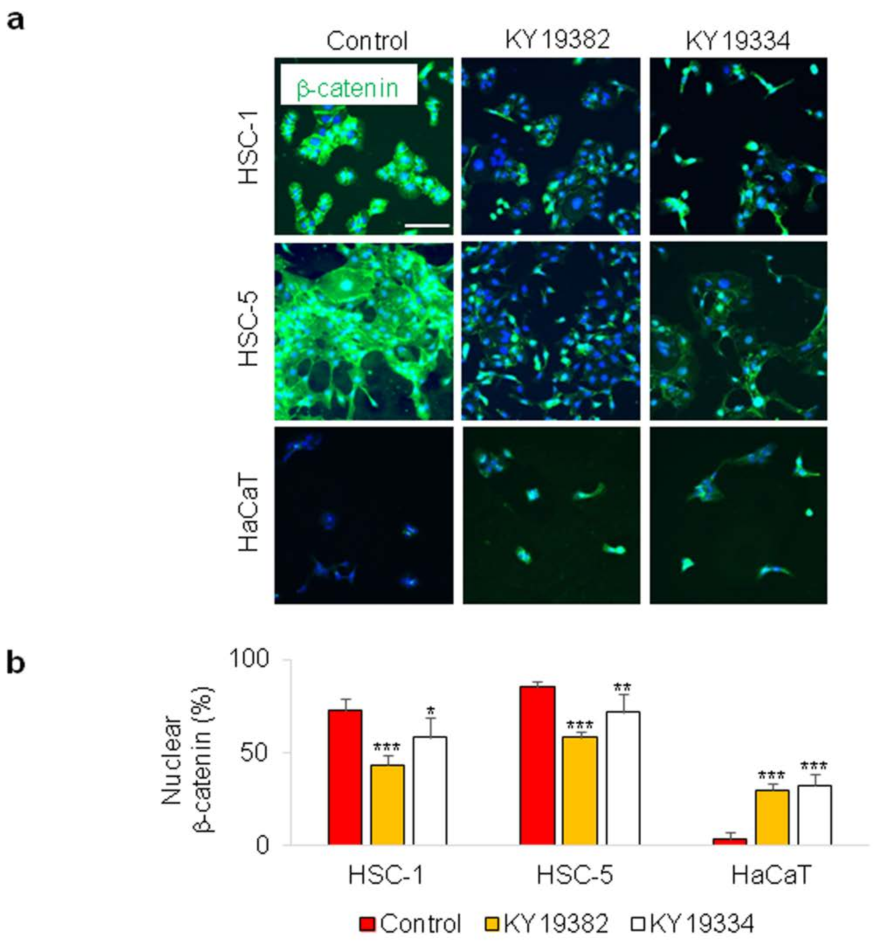


**Supplementary Fig. 2 Effects of KY19382 and KY19334 on Wnt activation in human cSCC cell lines. a** Immunocytochemical staining for β-catenin (green) and DAPI staining (blue) in HCS-1, HCS-5, and HaCaT cells treated with 5 μM of KY19382 or KY19334 for 24 h. Scale bar, 100 μm. **b** Quantification (*n* = 5) of β-catenin translocated to the nucleus in cells stained in Supplementary Fig. 2a. Data are represented as means ± SD. **P* <0.05, ***P* <0.01, ****P* <0.001 for panel **b**.

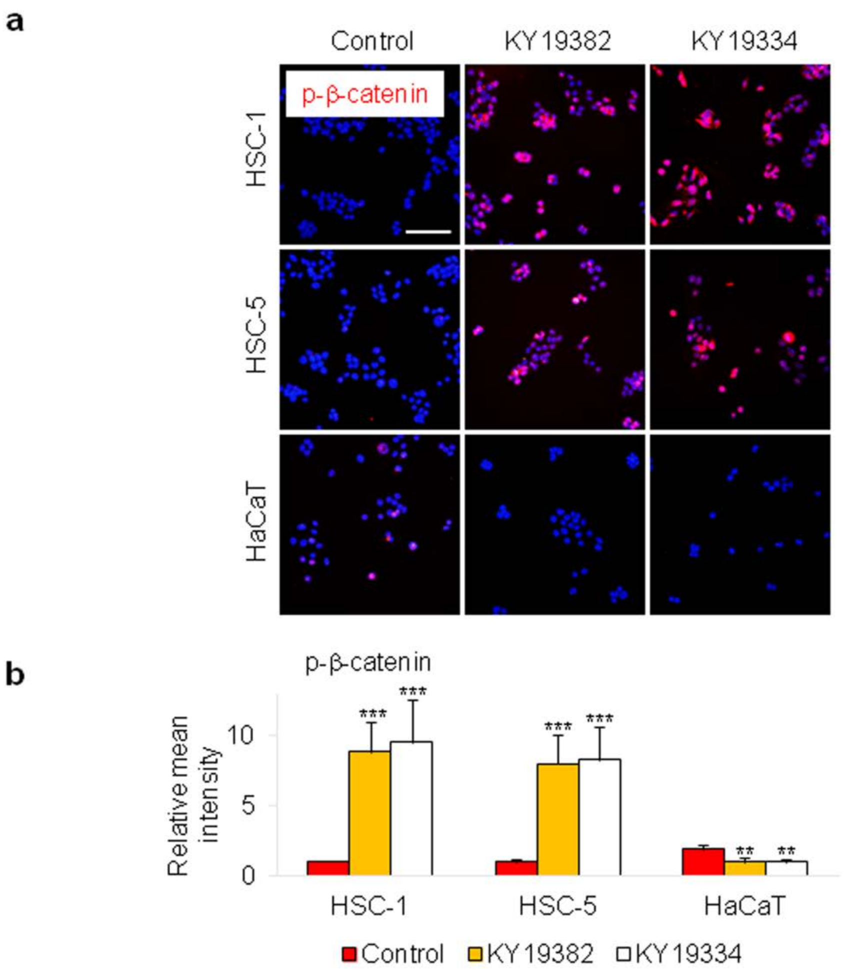


**Supplementary Fig. 3 Effects of KY19382 and KY19334 on β-catenin phosphorylation in human cSCC cell lines. a** Immunocytochemical staining for phospho-β-catenin (Ser33/37/Thr41) (red) and DAPI staining (blue) in HSC-1, HSC-5, and HaCaT cells treated with 5 μM KY19382 or KY19334 for 24 h. Scale bar, 100 μm. **b** Quantification (n = 5) of phospho-β-catenin levels in cells shown in Supplementary Fig. 3a. Data are presented as means ± SD. ***P* <0.01, ****P* <0.001 for panel **b**.

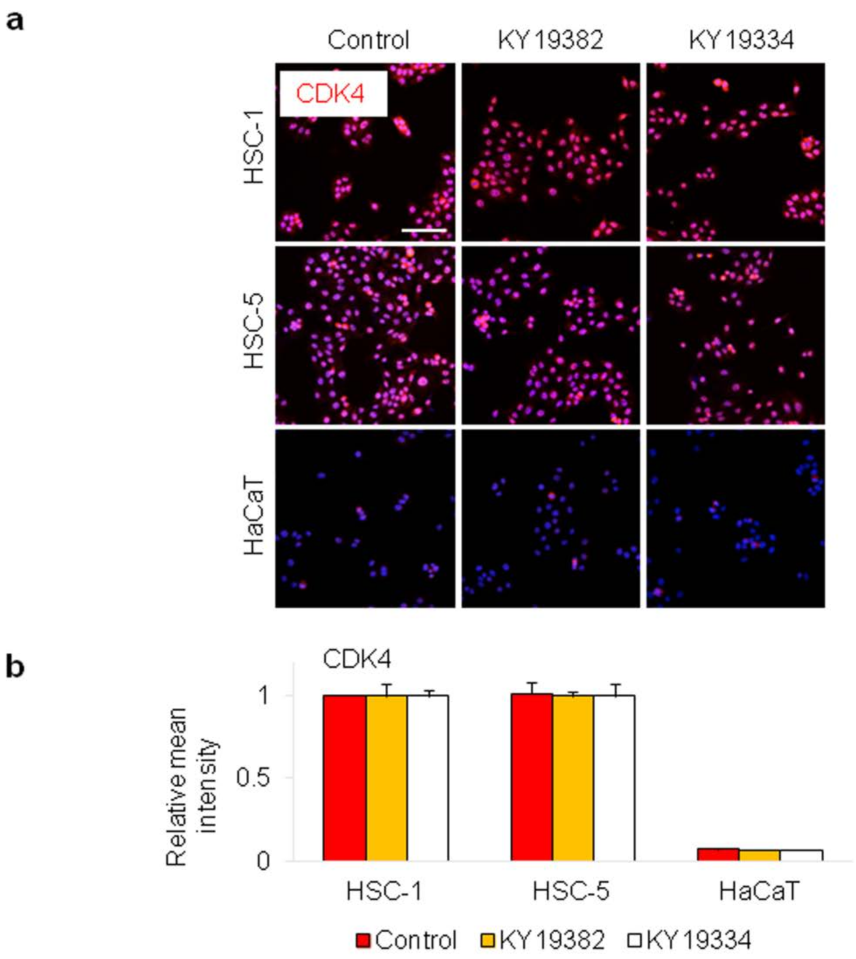


**Supplementary Fig. 4 Effects of KY19382 and KY19334 on CDK4 expression in human cSCC cell lines. a** Immunocytochemical staining for CDK4 (red) and DAPI staining (blue) in HSC-1, HSC-5, and HaCaT cells treated with 5 μM KY19382 or KY19334 for 24 h. Scale bar, 100 μm. **b** Quantification (n = 5) of CDK4 levels in cells shown in Supplementary Fig. 4a. Data are expressed as means ± SD. Absence of asterisks for panel **b**, indicating non-significant differences.

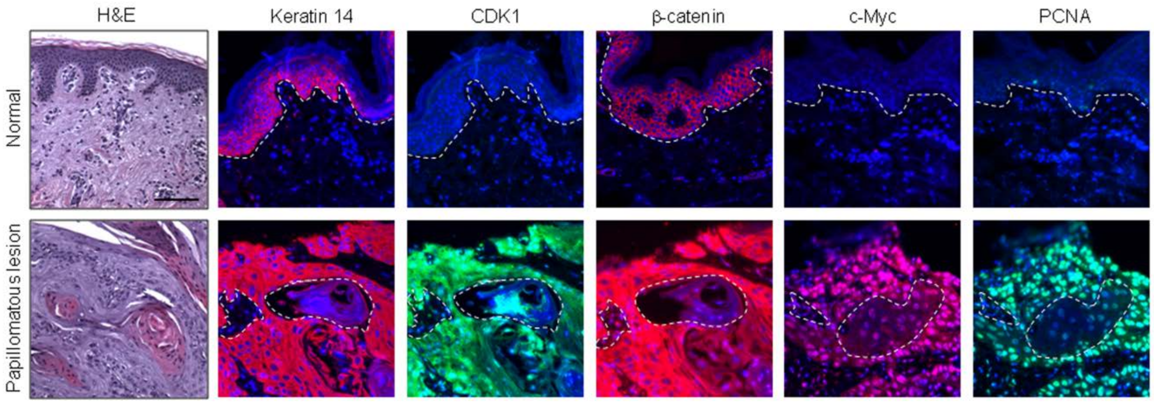


**Supplementary Fig. 5 Expression patterns of the CDK1 and β-catenin in human papillomatous lesion.** H&E and immunohistochemical staining for Keratin 14 (red), CDK1 (green), β-catenin (red), c-Myc (red), or PCNA (green) with DAPI nuclear counterstaining (blue) in normal and papillomatous human skin tissues. Dashed lines indicate the boundaries between K14-positive and negative tissues. Scale bar, 100 μm.

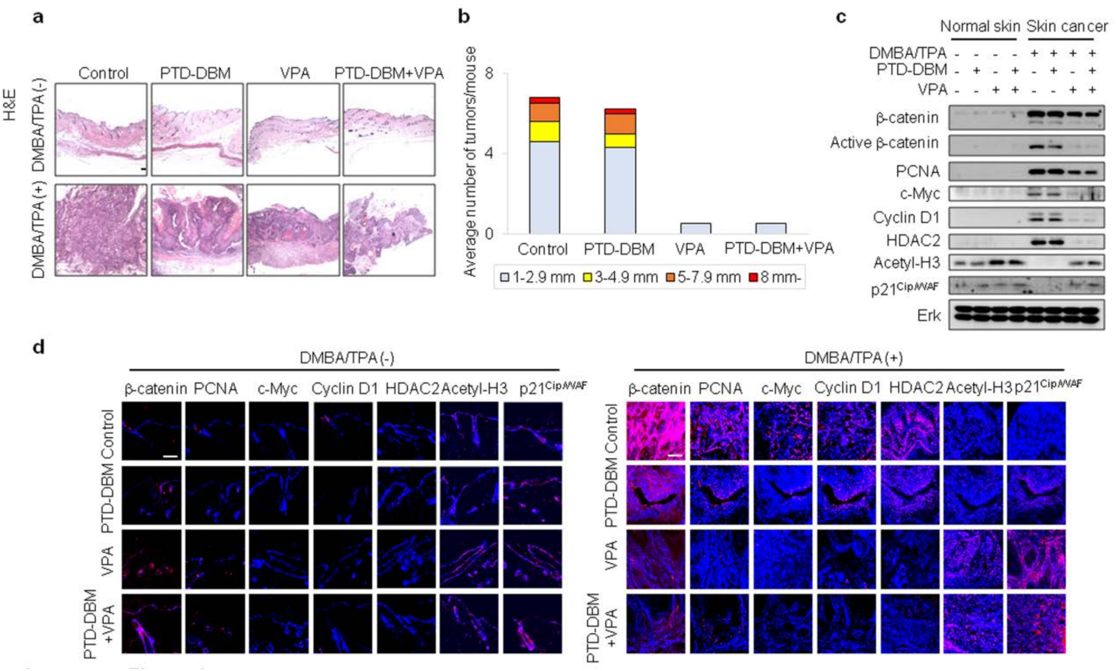


**Supplementary Fig. 6 Effects of PTD-DBM and/or VPA on cSCC development *in vivo*. a** H&E staining of normal skins and DMBA/TPA-induced tumors treated with 2 mM PTD-DBM and/or 500 mM VPA for 24 wk ($n$ = 10 per group). Scale bar, 100 μm. **b** Quantitative analyses ($n$ = 10 per group) of average number of DMBA/TPA-induced tumors treated with 2 mM PTD-DBM and/or 500 mM VPA for 24 wk by size. **c** Western blot analyses to detect protein levels of β-catenin, active β-catenin, Pcna, c-Myc, Cyclin D1, Hdac2, Acetyl-H3, p21$^{Cip/WAF}$, and Erk. **d** Immunohistochmical staining performed with antibodies against β-catenin, Pcna, c-Myc, Cyclin D1, Hdac2, Acetyl-H3, or p21$^{Cip/WAF}$, and DAPI staining (blue) in non-cancerous and cancerous mouse skin tissues treated with 2 mM PTD-DBM and/or 500 mM VPA for 24 wk. All primary antibodies were detected with Alexa 555-conjugated IgG secondary antibodies. Scale bar, 100 μm.

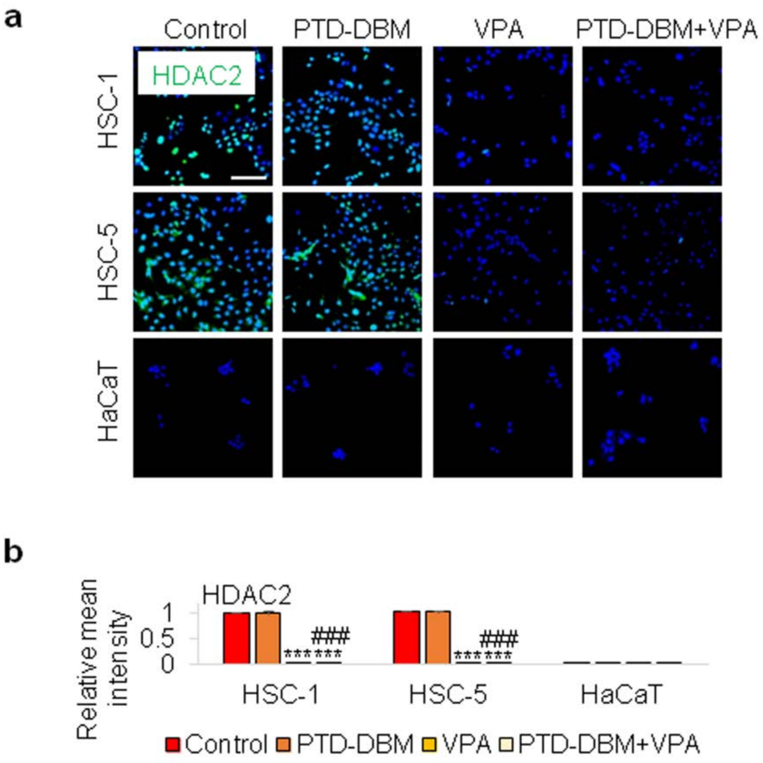


**Supplementary Fig. 7 Effects of VPA on HDAC2 inhibition in human cSCC cell lines. a** Immunocytochemical staining for HDAC2 (green), DAPI staining (blue) and mean intensity quantitation ($n$ = 5) in HSC-1, HSC-5, and HaCaT cells treated with 10 μM PTD-DBM and/or 2.5 mM VPA for 24 h. Scale bar, 100 μm. **b** Quantification ($n$ = 5) of HDAC2 in the cells stained in Supplementary Fig. 4a. Data are represented as means ± SD. ****P* <0.001 versus the control group; ###*P* <0.001 versus the PTD-DBM treatment group for panel **b**.

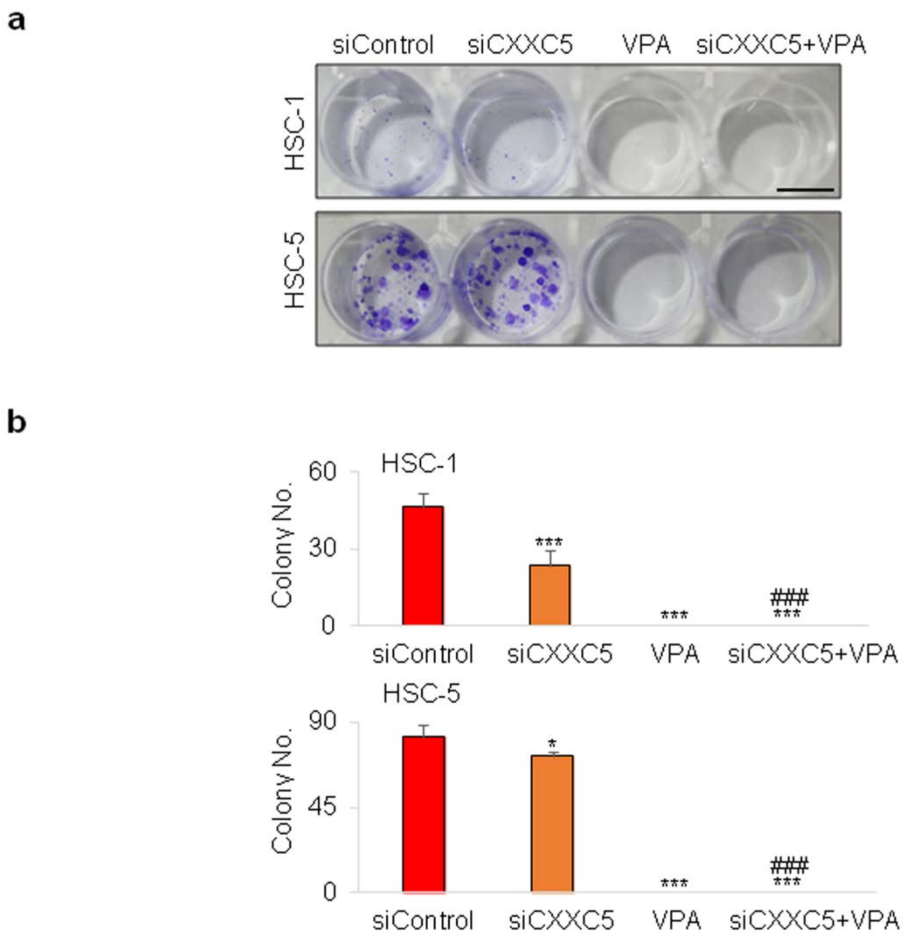


**Supplementary Fig. 8 Effects of CXXC5 knockdown and/or VPA treatment on colony formation in human cSCC cell lines. a** Representative images of cell colonies stained with crystal violet in HSC-1 and HSC-5 cells treated with 100 nM CXXC5 siRNA and/or 2.5 mM VPA for 2 wk. Scale bar, 1 cm. **b** Quantitative analyses of colonies formed in Supplementary Fig. 5a. Data are presented as means ± SD. **P* <0.05, ****P* <0.001 versus the control group; ###*P* <0.001 versus the CXXC5 knockdown group for panel **b**.

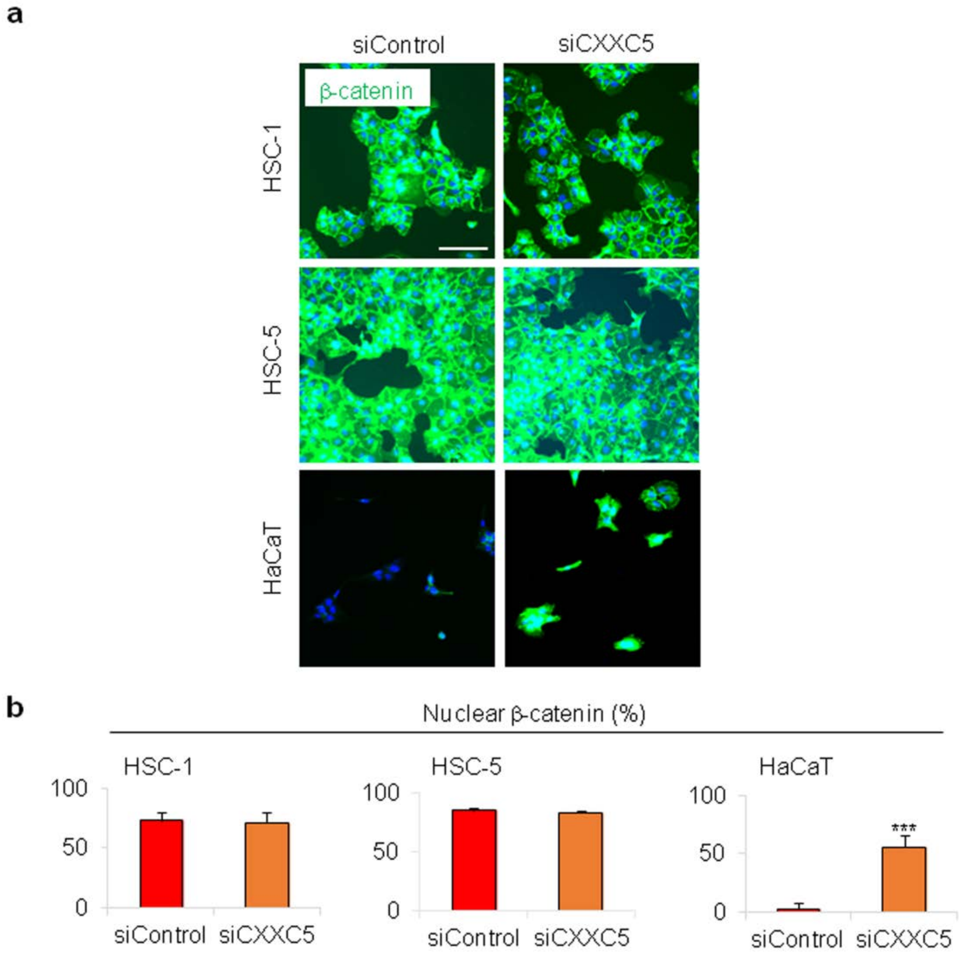


**Supplementary Fig. 9 Effects of CXXC5 knockdown on Wnt/β-catenin pathway activation in human cSCC cell lines. a** Immunocytochemical staining with antibody against β-catenin (green) and DAPI staining (blue) in HSC-1, HSC-5, and HaCaT cells 24 h after transfection with 100 nM of control siRNA or CXXC5 siRNA. Scale bar, 100 μm. **b** Quantitative analyses of nuclear β-catenin in the cells stained in Supplementary Fig. 6a. Data are represented as means ± SD. ***$P$ <0.001 for panel **b**.

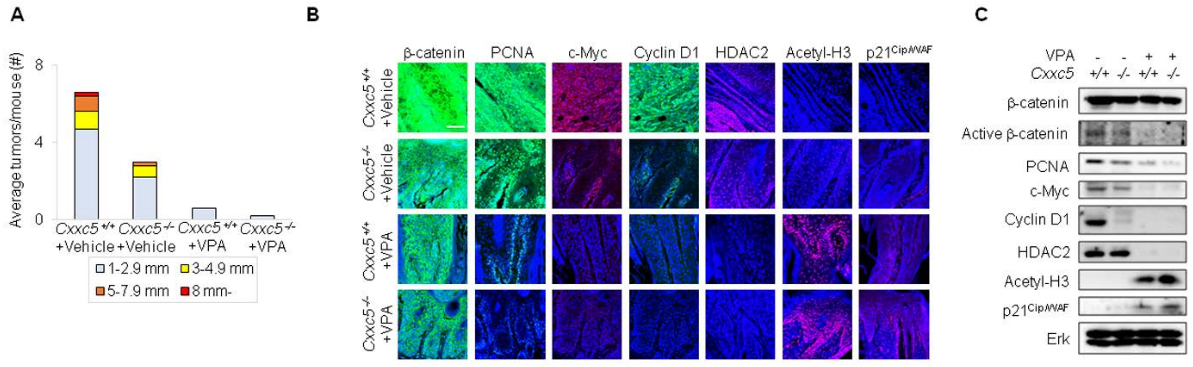


**Supplementary Fig. 10 Effects of CXXC5 knockout and/or VPA treatment on cSCC development *in vivo*. a** Quantitative analyses ($n$ = 10 per group) of average number of DMBA/TPA-induced tumors in *Cxxc5*$^{+/+}$ and *Cxxc5*$^{-/-}$ mice treated with or without 500 mM VPA for 24 wk by size. **b** Immunohistochmical staining performed with antibodies against β-catenin (green), Pcna (green), c-Myc (red), Cyclin D1 (green), Hdac2 (red), Acetyl-H3 (red), or p21$^{Cip/WAF}$ (red), and DAPI staining (blue) in DMBA/TPA-induced tumors arising in *Cxxc5*$^{+/+}$ and *Cxxc5*$^{-/-}$ mice treated with or without 500 mM VPA. Scale bar, 100 μm. **c** Western blot analyses to detect the protein levels of β-catenin, active β-catenin, Pcna, c-Myc, Cyclin D1, Hdac2, Acetyl-H3, p21$^{Cip/WAF}$, and Erk in DMBA/TPA-induced tumor tissues.
